# Completeness of Cancer Case Ascertainment in International Cancer Registries: Exploring the Issue of Gender Disparities

**DOI:** 10.3389/fonc.2020.01148

**Published:** 2020-07-16

**Authors:** Syed Ahsan Raza, Irfan Jawed, Roger Jamil Zoorob, Jason Lee Salemi

**Affiliations:** ^1^Department of Family and Community Medicine, Baylor College of Medicine, Houston, TX, United States; ^2^Department of Medicine, Section of Epidemiology and Population Sciences, Dan L. Duncan Comprehensive Cancer Center, Baylor College of Medicine, Houston, TX, United States; ^3^Houston Cancer Treatment Centers, Houston, TX, United States; ^4^College of Public Health, Morsani College of Medicine, University of South Florida, Tampa, FL, United States

**Keywords:** cancer, registry, case ascertainment, gender—, surveillance [methods]

## Introduction

Cancer case ascertainment is commonly called *case-finding* and is the process of identifying patients with malignant cancer who meet the inclusion criteria for a cancer registry. International cancer registries vary according to population size, funding, and trained personnel available for functioning. Most of these registries have strategic and logistical autonomy and follow their own standard registration procedures. The usefulness of population-based cancer registries across different geographic regions depends heavily on quality indices of registration and in particular, on *completeness* (1–6). Completeness is among the most important quality indicators of any cancer registry. It is defined as the *extent, degree or proportion of all incident cancer cases in a defined population that is included in the cancer registry database*. In theory, all cases of cancers in a defined population should be recorded in a population-based cancer registry or should be as close to 100% as possible (7, 8). In this opinion piece, we debate the issue of gender disparities along with rural-urban differences in the cancer registration process. Disparate methods of cancer case ascertainment in the registration process in men and women and their comparisions are also briefly discussed. We also suggest how the issue of gender disparities can be addressed through sex-ratio analysis of smoking associated cancer types by incorporating United Nations' Gender Inequality Index (GII). Because of subtle (and sometimes more elaborate) nuances, we have deliberately kept the terminology of “gender” and “sex” separate in our discussion such as *gender* disparity and *sex* ratios. The purpose of this discussion is to explore the issue of gender disparity in cancer registration and how this kind of potential bias can be recognized.

### Differences in Completeness of Ascertainment by Gender

Global collation of data on new cases of cancers through cancer registries provides an opportunity to explore gender differences in cancer incidence across diverse geographical regions (9). These differences are quite often interpreted in light of genetic and environmental causes of cancers across geographical regions (10, 11). Much has been written on gender disparities in specific types of cancer in both developed and resource-constrained parts of the world (12–18), yet there is a paucity of literature on differences in completeness of ascertainment (e.g., under-ascertainment) in cancer registries according to gender. Since the 1990s, there had been an increasing call to systematically quantify the completeness of cancer registries in the region(s) in which they operate (19–21). That call was heeded in the following decade, when studies on completeness of registration started appearing in literature from Africa and Eastern Europe (5, 22) and from developed parts of the world (23, 24). There are few studies that discussed or attempted to quantify the degree of under-reporting among women in a cancer registry (9, 25). Barlow et al. found overall under-reporting of 3.7% in a well-established Swedish Cancer Registry for the year of 1998 (25). In their study, there seemed to be a pattern of under-reporting that was worse in elderly women. Pearce et al. (9) concluded that the underlying socio-economic patterns of the community is important when interpreting incidence rates, especially among children from low-resource registries, where girls are more likely to be under-diagnosed.

Considering studies mentioned above, it is reasonable to suspect that in some resource-poor countries and conservative societies, due to socio-cultural dynamics, a female cancer patient may be more likely to be omitted from a cancer register. This can have important implications in the reporting and interpretation of incidence statistics and prevention strategies developed based on these data (11). It may be that women who are missed by registries are somewhat different from those who are identified, in terms of diagnostic or prognostic outcomes. It should also be noted that while this underestimation would still be present even if the women missed by registries are not different, there are also other artifacts to be considered that could affect the interpretation of incidence trends. These artifacts in interpreting incidence trends over time from cancer registries have been addressed by Saxem (26), Esteve (27), Muir (28), Swerdlow (29), and relatively recently by Bray (30). The required conditions that ensure truly valid comparisons of cancer incidence, as described by Muir et al. (28) [and quoted by Bray (30)], are worth repeating here unedited: (1) the definition and content of the cancer site being studied have not changed; (2) The criteria of malignancy have not changed; (3) the *likelihood* that a cancer will be diagnosed has not changed; (4) the progress of cancer from inception to diagnosis is not modified by early detection or screening programmes; (5) ascertainment of incident cases and deaths has been equally efficient throughout the period of study; (6) indexing in the International Classification of Diseases (ICD) has not changed; (7) accuracy and specificity of coding is consistent over time; (8) statistics are available at the level of detail required. These authors note, few, if any, databases would meet all of the above criteria. Comparisons of incidence rates of different cancer types between cancer registries under these kinds of a scenarios can therefore be biased, especially if there is also evidence of differences in the degree of under-ascertainment by gender.

### Gender Biases and Urban-Rural Gradient

Quantitative assessment of gender bias in registration was inferred using data from the Kampala Cancer Registry in Uganda by Templeton and Bianchi (31). Their publication in 1972 reported registration of women to be half as complete as those of men. However, they also reported that this bias in registration diminished as social patterns of literacy and health awareness evolved and when hospitals became more accessible (31, 32). Even if universal healthcare becomes a possibility in some low-resource countries and with improvements in overall cancer registration, coverage is not likely to be equal in both men and women (33). In addition to problems of health-care accessibility (more so reported in female patients), a cancer diagnosed in a hospital can also be influenced by age, tribal and ethnic affiliations, education, and social status in some countries (34).

Independent studies on cancer case-ascertainment from Bulgaria, Canada, Spain, Italy, India and Gambia have reported *level of completeness* by comparing commonly used indices of completeness (MV%: percent morphologically verified; DCO%: percent death certificate only; M:I Mortality-to-incidence ratio) in men and women (Table 1) (35–40). From among these studies, the Canadian registry (36) has shown better completeness indices relative to others in both genders. With the exception of the Gambian registry (40), these population-based registries are included in Cancer Incidence in Five Continents (CI-5) database of International Agency of Research on Cancer (IARC) (41). The Gambian study revealed heterogeneity in quality indicators, in particular, completeness, that suggested ascertainment issues in both genders. The study also reported lower incidence rates for several cancer types in both men and women in comparison with other West African cancer registries such as in Mali, Guinea, Cote d'Ivoire, Niger, and Nigeria (40, 42). According to the authors, the differences in cancer incidence rates between Gambians and other Africans may either represent true geographic variation in risk or there might be other factors at play. One factor was the registry's predominant coverage of the rural population of Gambia, and the related fact that other comparable registries in Africa were not rural. Just like gender disparities, this rural-urban contrast highlights several possible issues such as under-utilization of medical facilities in rural areas, under-diagnoses of cancer in low-resource rural health care settings, and under-reporting of cancer cases from rural populations by registry staff. Conversely, it is possible that it represents a true difference in the risk of cancer between rural and urban regions (in this case, a truly lower incidence in rural Gambia). A similar urban–rural difference in cancer incidence in both genders has been observed elsewhere (43, 44), and much of the difference was attributed to socio-economic deprivation.

**Table 1:** Completeness of cancer case-ascertainment for all ages in males and females using standard methods of ascertainment.

**PBCR**	**Authors, reference (year)**	**Male**			**Female**		
		**MV (%)**	**DCO (%)**	**M:I (%)**	**MV (%)**	**DCO (%)**	**M:I (%)**
Bulgaria	Dimitrova, (35) 2015	73.3	9.8	65.9	82.8	6.9	50.5
Canada	Zakaria, (36) 2013	90.0	0.9	48.8	90.0	1.2	48.5
Spain	Navarro, (37) 2010	88.7	2.6	52.3	87.8	3.8	48.0
Italy	Tumino, (38) 2004	83.0	2.0	54.0	85.0	3.0	48.0
India	Mathew, (39) 2011	83.2	1.4	12.6	81.5	1.1	9.3
Gambia	Shimakawa, (40) 2013	18.1	6.6	NR	33.1	3.6	NR

*MV%, percent morphologically verified; DCO%, percent death certificate only; M:I, Mortality-to-incidence ratio; NR, not reported. PBCR, Population Based Cancer Registries*.

Completeness of cancer case-ascertainment can therefore be confounded by gender effects in terms of access to cancer care services in urban-rural dynamics. Access to health care services are basic human rights and these rights are not always distributed equitably among men and women in many parts of the world (45, 46). Some of the studies are small-scale (47, 48), but they provide important insights into the experiences of women as they navigate the healthcare system. While the study of cancer care access by Sakellariou and Rotarou (46) focused on comparison among disabled and non-disabled women, their conclusion can be equally applied on the male-female differences in the access to health services (e.g., poor socioeconomic conditions of women and their lack of utilization of cancer care services). Gender effects studies (49, 50) have suggested that men receive more cancer detection tests than women in the same medical practices. Lack of access to health care services in some parts of the world give indication that there is indeed a possibility of a gender gap in cancer registration, but few studies exists that have actually embarked on exploring this issue in the field of cancer surveillance, with emphasis on the registration process itself (9, 51). Parker pointed out an exceptionally high cancer registration ratio (boys relative to girls) for childhood cancers in Afghanistan, Bangladesh, Morocco, Pakistan, and Papua New Guinea. He concluded that the striking gender-bias gives more information on socio-economic dynamics at play than on the etiology of the cancer (51).

## Methods of Case Ascertainment

In the past, several methods were used to assess completeness of case ascertainment in cancer registration (1, 2, 19, 49–54). Parkin and Bray have separated these methods into two broad categories (55): *Qualitative methods* give an indication of the degree of completeness relative to other registries, over time. Examples include historic data methods, percent of morphologically-verified cases (MV %), and mortality-to-incidence (M:I) ratios. *Quantitative methods* include “death certificate methods” and more sophisticated methods such as “capture-recapture” and the “Bullard-Flow” that provide a numerical evaluation of the extent to which all eligible cases are registered. Brief overview of these methods in terms of their uses and shortcomings are as follows:

### Morphological Verification (MV) of Diagnosis

Histological verification of cancer or “*Percent of cases morphologically verified (MV %),”* is a measure of the validity of the information and completeness in a registry (41). A very high proportion of cases diagnosed microscopically by histology or cytology/hematology (higher than reasonably expected) suggests over-reliance on the pathological laboratories as a source of information, and failure to find cancer cases diagnosed by other means. The percentage of cancer cases likely to be histologically verified for a given cancer type is dependent upon local regional circumstances where the registries are situated (52). It might be low if the means for taking biopsies, or examining the tissue, are lacking or inadequate such as in low resource countries (e.g., Gambia in Table 1).

### Mortality:Incidence (M:I) Ratios

The M:I ratio is a key indicator of completeness and involves comparison of the number of deaths (obtained from a source independent of the registry, e.g., the vital statistics system) and the number of incident cancer cases, registered in the same period (41). The M:I ratio may also reflect local conditions because survival and the quality of mortality statistics are at many levels related to the socioeconomic development of the region. Values of M:I over time that are greater than expected signals under-registration (i.e., incident cancers missed by the registry), and becomes more noticeable if this under-registration involves more than one type of cancer in a registry. However, under- or over-reporting of tumors on death certificates distorts this ratio, as will a lack of constancy in incidence and case fatality (the rate of death amongst incident cases) over time. Application of this indicator of ascertainment does require, however, mortality data of good quality (53), something not always possible in low-resource registries.

### Death Certificate Methods (DC Methods)

Death certificates are one of the main sources of information in a cancer registry in developed countries (54), and have three main uses in cancer registration: (1) as a complementary source of information on new cancer cases, (2) as a quality control assessment of both completeness and validity, and (3) for studies on survival of registered patients. DC methods cannot be readily applied to cancer registries from low- and medium-income countries (55). Methods used by Ajiki (56) [and quoted by Parkin (57) and Kamo (58)] explains death certificates as a means of capturing information on cases that were not registered during life. Although the DC method is not an ideal indicator of completeness of registration, an elevated proportion of cases diagnosed through this method does suggest some level of incompleteness.

### Comparison of Ascertainment Methods

**Figure 1:**
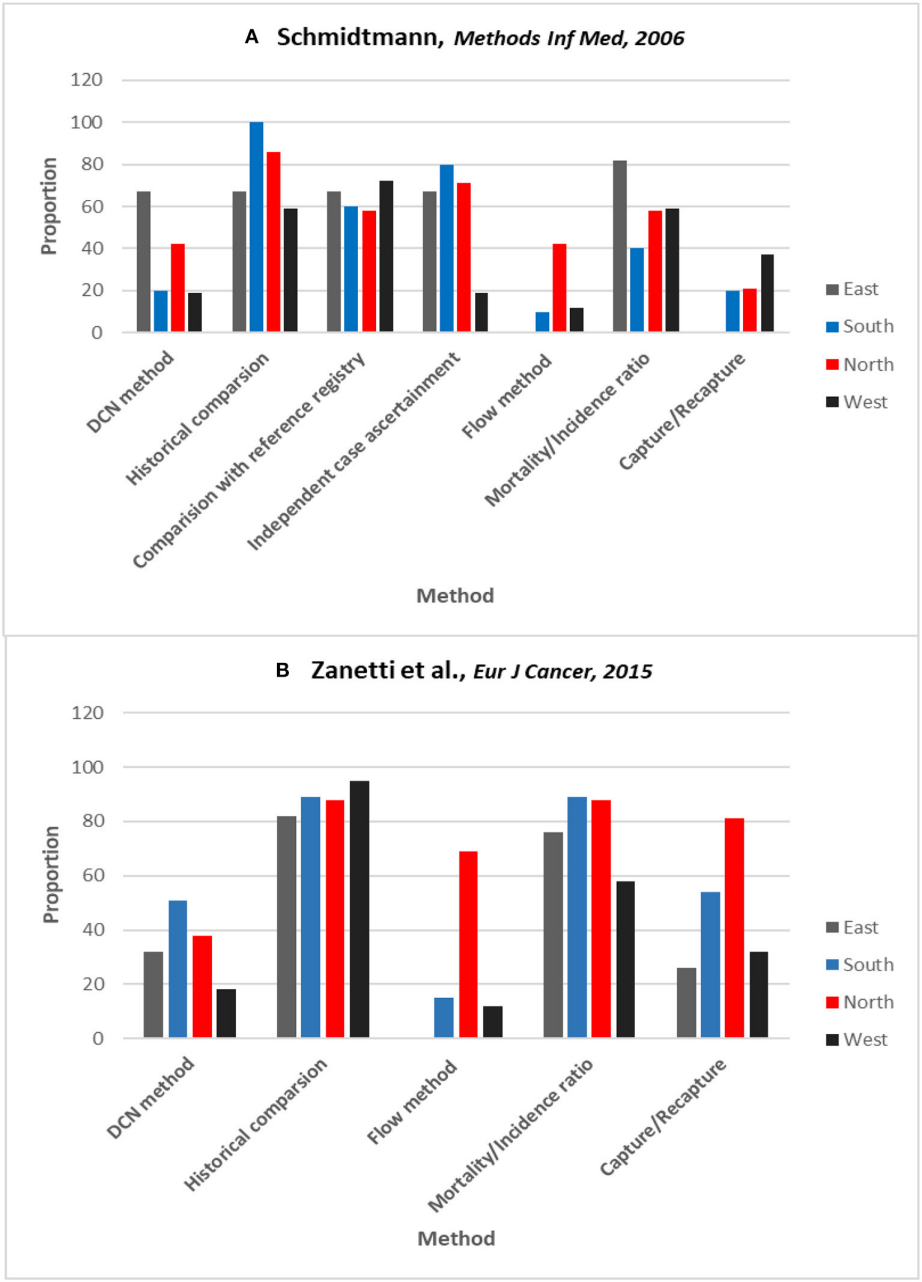
Surveys results on proportion of cancer registries within each region of Europe using different methods of estimating completeness. **(A)** Schmidtmann and Blettner. (60) **(B)** Zanetti et al. (61) in 2015. Countries grouped according to the definition of the UN Population Division (East, South, North, & West). DCN method is from where the no. of cases come from death certificates only (another term for DCO%). Reprinted with permission from authors and publishers (60, 61).

Commonly used indices (e.g., MV, DC, and M:I) as well as complex sophisticated methods [e.g., Bullard's Flow and capture-recapture method (1, 59)] are used in estimating the degree of completeness of ascertainment. This means that with the availability of various methods, the degree of completeness in cancer registries will vary with whatever methods are used. Schmidtmann and Blettner carried out the first survey of its kind to compare different methods that European cancer registries use to assess completeness of ascertainment (Figure 1A) (60). The study revealed that 86% of the 56 cancer registries that returned the survey questionnaire (of total of 195 registries that were contacted) had evaluated completeness of case ascertainment. The methods used most frequently were comparing current with historical incidence (73%) and comparisons with a presumably complete reference registry (65%). The M:I ratio was used in 58% of registries. More complex procedures, such as the capture-recapture method (25%) and Bullard's flow method (21%), were employed less often. The use of more than one method was also somewhat infrequent (29%). Zanetti et al. repeated the survey in 2015, with an improved response rate of 65% from cancer registries in Europe (Figure 1B) (61). The methods used were still largely based on simple indices with only slight improvement in the use of quantitative methods. The impression gained from these surveys is that there are different methods in use by individual cancer registries, and there are few comparative studies on their performance in relation to ascertainment in males and females. The authors of these studies suggest that in order to make valid comparisons across regions, modern registries should work more on standardizing methods of assessing completeness (60–62). These studies have underscored the importance of unifying methods for estimating completeness that could improve validity of incidence comparisons between cancer registries in both males and females.

## Identification of Gender Bias in Cancer Registration

In order to identify cancer registries with possible gender bias, we suggest a solution i.e., Sex Ratio analysis of cancer incidence that can circumvent some of the problems that exist in interpretations of incidence trends and their comparisons across different geographic areas. As a first step, one can compute sex ratios of different/particular types cancer incidence that can be carried out by identifying those cancer types from international cancer registries where the sex ratio has remained relatively stable (e.g., over time and geography). Secondly, this cancer specific sex ratio can be tallied to United Nations Gender Inequality Index (GII) to rank cancer registries according to their respective countries with low, moderate, and high gender inequalities over time. These categories of index, will help envisage how the stability of sex ratio compares with a geography where the registry is located e.g., a country with a uniquely high sex ratio of a cancer that has remained stably low over time in other regions can indicate bias in registration.

### Sex Ratio Analysis of Cancer Incidence

The proposed “Sex-Ratio Methodology” has opened new perspectives in disease epidemiology, specifically where the etiology remains undetermined or where new hypotheses are warranted, and old hypotheses can be confirmed (63–65). In fact, sex ratio is a robust epidemiological marker and its variability can be used for comparing data collected from different countries and regions, and where confounding effects exerted by different factors can be supposedly minimized (64, 66, 67). The sex ratio has also been recently used in cancer epidemiology using country-specific or worldwide cancer registries to speculate on causes of cancers (10, 68, 69).

### Using Gender Inequality Index

A well-recognized multidimensional indicator such as GII can be used in the context of exploring gender-bias in cancer registries (70). Completeness of cancer case ascertainment whether it is similar in males and females in international cancer registries can be explored through GII on selected cancer types that have remained stable over time. The measurement of gender inequality has received increasing attention over the past few years (71, 72) and has been explored in epidemiological studies (73, 74). The GII has been designed to capture gender inequality through relatively new functional form to summarize multidimensional information into a real number that can be used to compare countries' performance in this domain over time. The GII reflects gender-based disadvantage in three dimensions namely: reproductive health, empowerment and the labor market, for 160 countries. It shows the loss in potential human development due to inequality between male and female achievements in these three dimensions. It ranges from 0, where women and men fare equally, to 1, where one gender fares as poorly as possible in all measured dimensions (70). As of 2015 data, the lowest gender inequality country is Switzerland (GII: 0.04) and the highest gender inequality of 0.77 is found in Yemen (70). This type of analysis in conjunction with sex ratio of cancer incidence can also provide clues on quality of cancer registries and can inform the public health debate surrounding the contextual problem of gender-bias in cancer registration.

### Stable (and Variable) Sex Ratios of Cancer Incidence

To explore the issue of potential gender bias due to the possibility of differential disparities created by health seeking behaviors such as access to health care facilities and therapeutic treatment of cancers, we can select cancers that are somewhat known to be stable across time and geography e.g., kidney, leukemia, multiple myeloma, brain and possibly thyroid that varies to some extent (75–77). Hypothetical mock table (Table 2) presents cancer registries in countries that can be listed according to low and high gender inequality index with cancer types where the sex ratios of cancer incidence has been posited as relatively stable in the literature.

**Table 2:** Mock table for sex ratios of kidney, leukemia, multiple myeloma, brain, and thyroid cancers with gender inequality index (GII) values over time and their ranking.

**Registry (country)**	**GII values (World Rank)**	**Sex Ratios**				
		**Kidney**	**Leukemia**	**Multiple myeloma**	**Brain**	**Thyroid**
1	0.023 (1)	2.2	1.5	1.5	1.3	0.4
2	0.026 (3)	2.3	1.2	1.6	1.7	0.3
3	0.035 (5)	2.1	1.4	1.7	1.5	0.4
4	0.078 (6)	2.4	1.8	1.2	1.6	0.2
5	0.105 (7)	2.3	1.1	1.8	1.7	0.5
6	0.118 (10)	4.5	1.4	1.3	1.6	0.3
7	0.118 (11)	2.1	1.6	1.4	1.5	0.4
8	0.126 (12)	2.6	1.1	1.3	1.5	0.4
9	0.143 (18)	2.2	1.5	1.6	1.8	0.5
10	0.178 (29)	2.5	1.3	1.7	1.8	0.3
11	0.619 (119)	2.3	1.5	1.5	1.6	0.5
12	0.672 (135)	2.2	1.3	1.2	1.5	1.5
13	0.151 (145)	8.1	7.6	6.5	3.5	2.0
14	0.579 (154)	4.7	4.0	4.6	1.7	1.1

*Cancer registries in countries with lowest and highest gender inequality index (GII)*.

Table 2 also shows hypothetical world rankings of countries where the gender inequality is lowest (i.e., where females are likely to have equal access to health care services) e.g., registries 1–8 as well as where gender inequality is highest (registries 11 to 14). In reality, there are 160 countries of world with available GII values over time and rankings (70). Based on the index of gender inequality, it can also be assumed that gender bias can either be less or more of an issue in these cancer registries. For example, relatively similar values of sex ratios for five selected cancer types in Registry 1, 2, and 3 indicate that gender bias might be less of an issue in these registries because of stable sex ratios. One notable observation is Registry 6 where GII shows that it is a fairly gender balanced country in terms of perceived economic advantages and is ranked tenth. However, an extremely high sex ratios of 4.5 in Registry 6 (for kidney cancer) is indicative that the male and female completeness of ascertainment (and other artifacts) might not be similar (i.e., more males are registered than females). Registry 6 is also showing that it is specific for cancer of kidney whereas sex ratios of other cancer types are stable compared to other registries. High GII countries with Registries 13 and 14 also provide evidence of major quality issues in registration process. Hence gender bias can be indicated if we find these kinds of discrepancies in registries located in countries with either low, moderate or high GII.

## Conclusions

In summary, this opinion piece highlights contextual problems that underlie disparities in completeness of ascertainment by gender in cancer registries around the world. Implementing protocols for assessing the completeness of ascertainment by person, place, and time is invaluable in providing clues to the relative quality of cancer registries. Cancer cases can only be recorded once they have been diagnosed, after a patient has sought medical attention. It is possible that in rural areas of developing countries, people can die with their cancer before ever having been seen by a medical doctor. This is less likely to be common in the more urban populations of the twenty-first century (58). In some countries, cancer registration has a legal basis and is funded by governments, but some registries, particularly in developing countries, have operated on a voluntary basis, relying on good will and the tradition of sharing of medical information among different medical specialties (59). Notwithstanding the existence of contextual obstacles in cancer registration, population-based cancer registries do provide a good source of information to study the causes of cancers (37, 60, 61). When we can begin to quantify potential biases in ascertainment across population subgroups (e.g., by gender), we can improve the utility of these data.

## Author Contributions

SR carried out the literature review and wrote the first draft of the manuscript. IJ, RZ, and JS provided critical revisions of the manuscript and ensured accurate interpretation of the evidence. All authors contributed to the article and approved the submitted version.

## Conflict of Interest

The authors declare that the research was conducted in the absence of any commercial or financial relationships that could be construed as a potential conflict of interest.

## References

[1] Bullard J Coleman MP Robinson D Lutz JM Bell J Peto J. Completeness of cancer registration: a new method for routine use. Br J Cancer. (2000) 82:1111–6. 10.1054/bjoc.1999.1048 10737395 PMC2374436

[2] Das B Clegg LX Feuer EJ Pickle LW. A new method to evaluate the completeness of case ascertainment by a cancer registry. Cancer Causes Control. (2008) 19:515–25. 10.1007/s10552-008-9114-0 18270798 PMC2668648

[3] Inoue M Tajima K Inuzuka K Tominaga S. The estimation of cancer incidence in Aichi Prefecture, Japan: use of degree of completeness of registration. J Epidemiol. (1998) 8:60–4. 10.2188/jea.8.60 9575697

[4] Jedy-Agba EE Curado MP Oga E Samaila MO Ezeome ER Obiorah C. The role of hospital-based cancer registries in low and middle income countries-The Nigerian Case Study. Cancer Epidemiol. (2012) 36:430–5. 10.1016/j.canep.2012.05.010 22704971 PMC3438360

[5] Parkin DM Wabinga H Nambooze S. Completeness in an African cancer registry. Cancer Causes Control. (2001) 12:147–52. 10.1023/A:1008966225984 11246843

[6] Suwanrungruang K Sriplung H Attasara P Temiyasathit S Buasom R Waisri N. Quality of case ascertainment in cancer registries: a proposal for a virtual three-source capture-recapture technique. Asian Pac J Cancer Prev. (2011) 12:173−8. 21517253

[7] Gail M Benichou J Encyclopedia of Epidemiologic Methods. Chichester: Wiley (2000). p. 125–9.

[8] Powell J Cancer registration: principles and methods. Data sources and reporting. IARC Sci Publ. (1991) 95:29–42. 1894330

[9] Pearce MS Parker L. Childhood cancer registrations in the developing world: still more boys than girls. Int J Cancer. (2001) 91:402–6. 10.1002/1097-0215(200002)9999:9999<::AID-IJC1048>3.0.CO;2-F 11169966

[10] Edgren G Liang L Adami HO Chang ET. Enigmatic sex disparities in cancer incidence. Eur J Epidemiol. (2012) 27:187–96. 10.1007/s10654-011-9647-5 22212865

[11] Rutherford MJ Moller H Lambert PC. A comprehensive assessment of the impact of errors in the cancer registration process on 1- and 5-year relative survival estimates. Br J Cancer. (2013) 108:691–8. 10.1038/bjc.2013.12 23361055 PMC3593558

[12] Burge F Kockelbergh R. Closing the gender gap: can we improve bladder cancer survival in women? - A systematic review of diagnosis, treatment and outcomes. Urol Int. (2016) 97:373–9. 10.1159/000449256 27595416

[13] Dominguez-Gordillo A Esparza-Gomez G Garcia-Jimenez B Cerero-Lapiedra R Casado-Gomez I Romero-Lastra P. The pattern of lip cancer occurrence over the 1990-2011 period in public hospitals in Madrid, Spain. J Oral Pathol Med. (2016) 45:202–10. 10.1111/jop.12340 26256568

[14] Fenner A. Bladder cancer: bridging the gender gap in bladder cancer diagnoses. Nat Rev Urol. (2013) 10:127. 10.1038/nrurol.2013.10 23381596

[15] Henning A Wehrberger M Madersbacher S Pycha A Martini T Comploj E. Do differences in clinical symptoms and referral patterns contribute to the gender gap in bladder cancer? BJU Int. (2013) 112:68–73. 10.1111/j.1464-410X.2012.11661.x 23320798

[16] Juneja A Adhikari T Pandey A Sharma S Sehgal A. Share of tobacco related cancers: gender and time gaps-Indian scenario. J Clin Diagn Res. (2015) 9:LC01–3. 10.7860/JCDR/2015/9912.5422 25738010 PMC4347101

[17] Pang HH Wang X Stinchcombe TE Wong ML Cheng P Ganti AK. Enrollment trends and disparity among patients with lung cancer in national clinical trials, 1990 to 2012. J Clin Oncol. (2016) 34:3992–9. 10.1200/JCO.2016.67.7088 27646951 PMC5477832

[18] Tabatabai MA Kengwoung-Keumo JJ Oates GR Guemmegne JT Akinlawon A Ekadi G. Racial and gender disparities in incidence of lung and bronchus cancer in the United States: a longitudinal analysis. PLoS ONE. (2016) 11:e0162949. 10.1371/journal.pone.0162949 27685944 PMC5042522

[19] Brewster D. Improving the quality of cancer registration data. J R Soc Med. (1995) 88:268–71. 7636820 PMC1295197

[20] Lapham R Waugh NR. An audit of the quality of cancer registration data. Br J Cancer. (1992) 66:552–4. 10.1038/bjc.1992.312 1520592 PMC1977955

[21] Swerdlow AJ dos Santos Silva I Reid A Qiao Z Brewster DH Arrundale J. Trends in cancer incidence and mortality in Scotland: description and possible explanations. Br J Cancer. (1998) 77 (Suppl. 3):1–54. 10.1038/bjc.1998.424 9665378 PMC2149878

[22] Lang K Magi M Aareleid T. Study of completeness of registration at the Estonian cancer registry. Eur J Cancer Prev. (2003) 12:153–6. 10.1097/00008469-200304000-00009 12671539

[23] Dickinson HO Salotti JA Birch PJ Reid MM Malcolm A Parker L. How complete and accurate are cancer registrations notified by the National Health Service Central Register for England and Wales? J Epidemiol Community Health. (2001) 55:414–22. 10.1136/jech.55.6.414 11351000 PMC1731913

[24] Tingulstad S Halvorsen T Norstein J Hagen B Skjeldestad FE. Completeness and accuracy of registration of ovarian cancer in the cancer registry of Norway. Int J Cancer. (2002) 98:907–11. 10.1002/ijc.10254 11948471

[25] Barlow L Westergren K Holmberg L Talback M. The completeness of the Swedish Cancer Register: a sample survey for year 1998. Acta Oncol. (2009) 48:27–33. 10.1080/02841860802247664 18767000

[26] Saxen E Trends: Facts or Fallacy. Trends in Cancer Incidence: Causea and Practical Implications. Oslo: The International Union Against Cancer and The Norwegian Cancer Society (1982). p. 5–16.

[27] Esteve J. International study of time trends. Some methodological considerations. Ann N Y Acad Sci. (1990) 609:77–84; discussion 84–6. 10.1111/j.1749-6632.1990.tb32058.x 2264659

[28] Muir CS Fraumeni JF Jr Doll R The interpretation of time trends. Cancer Surv. (1994) 19–20:5–21. 7895222

[29] Swerdlow AJ Douglas AJ Vaughan Hudson G Vaughan Hudson B. Completeness of cancer registration in England and Wales: an assessment based on 2,145 patients with Hodgkin's disease independently registered by the British National Lymphoma Investigation. Br J Cancer. (1993) 67:326–9. 10.1038/bjc.1993.60 8431361 PMC1968180

[30] Bray F. The evolving scale and profile of cancer worldwide: much ado about everything. Cancer Epidemiol Biomarkers Prev. (2016) 25:3–5. 10.1158/1055-9965.EPI-15-1109 26667885

[31] Templeton AC Bianchi A. Bias in an African cancer registry. Int J Cancer. (1972) 10:186–93. 10.1002/ijc.2910100124 4661562

[32] Templeton AC Buxton E Bianchi A. Cancer in Kyadondo County, Uganda, 1968-70. J Natl Cancer Inst. (1972) 48:865–74. 5023685

[33] Binagwaho A Farmer PE Nsanzimana S Karema C Gasana M de Dieu Ngirabega J. Rwanda 20 years on: investing in life. Lancet. (2014) 384:371–5. 10.1016/S0140-6736(14)60574-2 24703831 PMC4151975

[34] Swaminathan R Sankaranarayanan R. Under-diagnosis and under-ascertainment of cases may be the reasons for low childhood cancer incidence in rural India. Cancer Epidemiol. (2010) 34:107–8. 10.1016/j.canep.2009.11.006 20022839

[35] Dimitrova N Parkin DM. Data quality at the Bulgarian National Cancer Registry: An overview of comparability, completeness, validity and timeliness. Cancer Epidemiol. (2015) 39:405–13. 10.1016/j.canep.2015.03.015 25914137

[36] Zakaria D. An examination of the NAACCR method of assessing completeness of case ascertainment using the Canadian Cancer Registry. Health Rep. (2013) 24:3–13. 24258322

[37] Navarro C Martos C Ardanaz E Galceran J Izarzugaza I Peris-Bonet R. Population-based cancer registries in Spain and their role in cancer control. Ann Oncol. (2010) 21 (Suppl. 3):iii3–13. 10.1093/annonc/mdq094 20427357

[38] Tumino R Ferretti S. Quality and completeness indices. Epidemiol Prev. (2004) 28 (2 Suppl.):17–21. 15281601

[39] Mathew A Daniel CR Ferrucci LM Seth T Devesa SS George PS. Assessment of follow-up, and the completeness and accuracy of cancer case ascertainment in three areas of India. Cancer Epidemiol. (2011) 35:334–41. 10.1016/j.canep.2011.03.006 21621499 PMC3460518

[40] Shimakawa Y Bah E Wild CP Hall AJ. Evaluation of data quality at the Gambia national cancer registry. Int J Cancer. (2013) 132:658–65. 10.1002/ijc.27646 22618962

[41] Forman D Bray F Brewster DH Gombe Mbalawa C Kohler B Piñeros M. Cancer Incidence in Five Continents, Vol. X. Lyon: International Agency for Research on Cancer (2014). 10.1002/ijc.29670 26135522

[42] Bah E Parkin DM Hall AJ Jack AD Whittle H. Cancer in the Gambia: 1988-97. Br J Cancer. (2001) 84:1207–14. 10.1054/bjoc.2001.1730 11336472 PMC2363873

[43] Monroe AC Ricketts TC Savitz LA. Cancer in rural versus urban populations: a review. J Rural Health. (1992) 8:212–20. 10.1111/j.1748-0361.1992.tb00354.x 10121550

[44] Swaminathan R Selvakumaran R Esmy PO Sampath P Ferlay J Jissa V. Cancer pattern and survival in a rural district in South India. Cancer Epidemiol. (2009) 33:325–31. 10.1016/j.canep.2009.09.008 19853553

[45] Mandelblatt JS Yabroff KR Kerner JF. Equitable access to cancer services: a review of barriers to quality care. Cancer. (1999) 86:2378–90. 10.1002/(SICI)1097-0142(19991201)86:11<2378::AID-CNCR28>3.0.CO;2-L 10590381

[46] Sakellariou D Rotarou ES. Utilisation of cancer screening services by disabled women in Chile. PLoS ONE. (2017) 12:e0176270. 10.1371/journal.pone.0176270 28459874 PMC5411071

[47] Peters K Cotton A. Barriers to breast cancer screening in Australia: experiences of women with physical disabilities. J Clin Nurs. (2015) 24:563–72. 10.1111/jocn.12696 25236777

[48] Ramjan L Cotton A Algoso M Peters K. Barriers to breast and cervical cancer screening for women with physical disability: a review. Women Health. (2016) 56:141–56. 10.1080/03630242.2015.1086463 26325597

[49] McCusker J Morrow GR. Factors related to the use of cancer early detection techniques. Prev Med. (1980) 9:388–97. 10.1016/0091-7435(80)90233-9 7208447

[50] Womeodu RJ Bailey JE. Barriers to cancer screening. Med Clin North Am. (1996) 80:115–33. 10.1016/S0025-7125(05)70430-2 8569291

[51] Parker L. Children's cancer in the developing world: where are the girls? Pediatr Hematol Oncol. (1998) 15:99–103. 10.3109/08880019809167223 9592835

[52] Bray F Ferlay J Laversanne M Brewster DH Gombe Mbalawa C Kohler B. Cancer incidence in five continents: inclusion criteria, highlights from Volume X and the global status of cancer registration. Int J Cancer. (2015) 137:2060–71. 10.1002/ijc.29670 26135522

[53] Colonna M Grosclaude P Faivre J Revzani A Arveux P Chaplain G. Cancer registry data based estimation of regional cancer incidence: application to breast and colorectal cancer in French administrative regions. J Epidemiol Community Health. (1999) 53:558–64. 10.1136/jech.53.9.558 10562880 PMC1756959

[54] Castro C Bento MJ Lunet N Campos P. Assessing the completeness of cancer registration using suboptimal death certificate information. Eur J Cancer Prev. (2012) 21:478–9. 10.1097/CEJ.0b013e32834f811c 22193365

[55] Parkin DM Chen VW Ferlay J Galceran J Storm H Whelan S. Comparability and Quality Control in Cancer Registration. (IARC Technical Report No. 19). Lyon: IARC (WHO) and IACR (1994).

[56] Ajiki W Tsukuma H Oshima A. [Index for evaluating completeness of registration in population-based cancer registries and estimation of registration rate at the Osaka Cancer Registry between 1966 and 1992 using this index]. Nihon Koshu Eisei Zasshi. (1998) 45:1011–7. 9893469

[57] Parkin DM Bray F. Evaluation of data quality in the cancer registry: principles and methods Part II. Completeness. Eur J Cancer. (2009) 45:756–64. 10.1016/j.ejca.2008.11.033 19128954

[58] Kamo K Kaneko S Satoh K Yanagihara H Mizuno S Sobue T. A mathematical estimation of true cancer incidence using data from population-based cancer registries. Jpn J Clin Oncol. (2007) 37:150–5. 10.1093/jjco/hyl143 17272318

[59] Crocetti E Miccinesi G Paci E Zappa M. An application of the two-source capture-recapture method to estimate the completeness of the Tuscany Cancer Registry, Italy. Eur J Cancer Prev. (2001) 10:417–23. 10.1097/00008469-200110000-00005 11711756

[60] Schmidtmann I Blettner M. How do cancer registries in europe estimate completeness of registration? Methods Inf Med. (2009) 48:267–71. 10.3414/ME0559 19387506

[61] Zanetti R Schmidtmann I Sacchetto L Binder-Foucard F Bordoni A Coza D. Completeness and timeliness: cancer registries could/should improve their performance. Eur J Cancer. (2015) 51:1091–8. 10.1016/j.ejca.2013.11.040 24393522

[62] Schmidtmann I. Estimating completeness in cancer registries–comparing capture-recapture methods in a simulation study. Biom J. (2008) 50:1077–92. 10.1002/bimj.200810483 19067337

[63] Palacios N Alonso A Bronnum-Hansen H Ascherio A. Smoking and increased risk of multiple sclerosis: parallel trends in the sex ratio reinforce the evidence. Ann Epidemiol. (2011) 21:536–42. 10.1016/j.annepidem.2011.03.001 21550815 PMC3124940

[64] Trojano M Lucchese G Graziano G Taylor BV Simpson S Jr. Geographical variations in sex ratio trends over time in multiple sclerosis. PLoS ONE. (2012) 7:e48078. 10.1371/journal.pone.0048078 23133550 PMC3485003

[65] Zhao J Booth H Dear K Tu EJ. Cardiovascular mortality sex differentials in selected East Asian and Western populations. J Epidemiol Community Health. (2016) 70:983–9. 10.1136/jech-2015-206577 27048151

[66] Lawlor DA Ebrahim S Davey Smith G. Sex matters: secular and geographical trends in sex differences in coronary heart disease mortality. BMJ. (2001) 323:541–5. 10.1136/bmj.323.7312.541 11546699 PMC48158

[67] Orton SM Herrera BM Yee IM Valdar W Ramagopalan SV Sadovnick AD. Sex ratio of multiple sclerosis in Canada: a longitudinal study. Lancet Neurol. (2006) 5:932–6. 10.1016/S1474-4422(06)70581-6 17052660

[68] Cook MB Dawsey SM Freedman ND Inskip PD Wichner SM Quraishi SM. Sex disparities in cancer incidence by period and age. Cancer Epidemiol Biomarkers Prev. (2009) 18:1174–82. 10.1158/1055-9965.EPI-08-1118 19293308 PMC2793271

[69] Cook MB McGlynn KA Devesa SS Freedman ND Anderson WF. Sex disparities in cancer mortality and survival. Cancer Epidemiol Biomarkers Prev. (2011) 20:1629–37. 10.1158/1055-9965.EPI-11-0246 21750167 PMC3153584

[70] Gender Inequality Index United Nations Devlopment Programme: Human Development Reports. Available online at: http://hdr.undp.org/en/content/gender-inequality-index-gii (accessed March 31, 2019).

[71] Permanyer I The measurement of multidimensional gender inequality: continuing the debate. Soc Indic Res. (2010) 95:181–98. 10.1007/s11205-009-9463-4

[72] Permanyer I A critical assessment of the UNDP's gender inequality index. Femin Econ. (2013) 19:1–32. 10.1080/13545701.2013.769687

[73] Hassanzadeh JMN Esmailnasab N Rezaeian S Bagheri P Armanmehr V The correlation between gender inequalities and their health related factors in world countries: a global cross-sectional study. Epidemiol Res Int. (2014):1–8. 10.1155/2014/521569

[74] Singh GK Azuine RE Siahpush M. Global inequalities in cervical cancer incidence and mortality are linked to deprivation, low socioeconomic status, and human development. Int J MCH AIDS. (2012) 1:17–30. 10.21106/ijma.12 27621956 PMC4948158

[75] Fallah M Kharazmi E. A method to adjust for ascertainment bias in the evaluation of cancer registry data. Asian Pac J Cancer Prev. (2007) 8:113–8. 17477784

[76] Scelo G Hofmann JN Banks RE Bigot P Bhatt RS Cancel-Tassin G. International cancer seminars: a focus on kidney cancer. Ann Oncol. (2016) 27:1382–5. 10.1093/annonc/mdw186 27130845 PMC4959923

[77] Scelo G Li P Chanudet E Muller DC. Variability of sex disparities in cancer incidence over 30 years: the striking case of kidney cancer. Eur Urol Focus. (2018) 4:586–90. 10.1016/j.euf.2017.01.006 28753845

